# An Exploration of Language and Communication Profiles in a Clinical Sample of People with Foetal Alcohol Spectrum Disorder (FASD) Seen in the UK National FASD Clinic: A Service Audit Comparing and Contrasting the Clinical Evaluation of Language Fundamentals (CELF), Children’s Communication Checklist (CCC) and Vineland Adaptive Behaviour Scales (VABS) Assessment Measures

**DOI:** 10.3390/children13050611

**Published:** 2026-04-28

**Authors:** Freya Morris, Alexandra Carlisle, Penny A. Cook, Lucy Deeprose, Bethany Dubbey, Louise Fox, Alexandra Livesey, Raja A. S. Mukherjee

**Affiliations:** 1 National FASD Clinic, Surrey and Borders Partnership NHS Foundation Trust, Gatton Place, St. Matthews Rd., Redhill RH1 1TA, UK; freya.morris@sabp.nhs.uk (F.M.);; 2 School of Health and Society, Mary Seacole Building, Frederick Road Campus, Broad St., Salford M6 6PU, UK; 3 School of Medicine, Faculty of Health and Medical Sciences, University of Surrey, Kate Granger Building, 20 Priestley Rd., Surrey Research Park, Guildford GU2 7HY, UK

**Keywords:** FASD, language, communication, testing

## Abstract

**Highlights:**

FASD is known to have communication as an area of core difficulty. Assessing this domain has been challenging outside of specialist services. This paper demonstrates that the CCC can be used by clinical pathways to evaluate this domain effectively even in the absence of a Specialist Speech and Language Therapist.

**What are the main findings?**

- The CCC is effective at evaluating language in people with FASD.
- The CCC showed significant correlations with the CELF in people with FASD.

**What is the implication of the main findings?**

- Clinics without access to a SALT can still evaluate language using the CCC.
- The CCC can be a cost effective evaluation of language for many resource restricted clinical settings.

**Abstract:**

Background/Objectives: Language difficulties have been recognised as common in people with FASD. Assessment of the language domain is important, and the CELF, CCC, and VABS are three commonly used language assessment tools. The CCC and VABS are both informant-report questionnaires, whilst the CELF is completed in person by a Speech and Language Therapist (SaLT). As many neurodevelopmental assessment clinics may not have access to a SaLT, the aim of this paper was to establish potential correlations between these measures in individuals with FASD, as well as to establish the clinical utility of questionnaire-based measures for assessment of communication in FASD in the absence of a multidisciplinary team. Methods: Data was drawn from the UK National FASD Clinic database containing 385 patient assessments. Sample size varied between 77 and 274 completed assessments depending on the measure due to change in clinical practice over several years. A two-tailed Spearman’s Correlations between CCC subscales and CELF domains were carried out. Two-tailed Spearman’s Correlations between the Communication domain of the VABS and the CELF were also conducted. Results: Significant correlations were found between all assessed CELF domains and the CCC Semantics and Coherence subdomains and GCC, as well as between the CCC Syntax subdomain and 3 of the CELF domains. No significant correlations were found between CCC Speech and any CELF domains. A mix of significant correlations between the VABS domain and subdomains and the CELF domains were found. Conclusions: These results suggest the potential clinical utility of using the CCC as a measure of an individual’s language functioning as part of an FASD assessment, particularly in the absence of access to direct assessment carried out by a SaLT. Future research should focus on the development of a questionnaire-based assessment battery for an FASD population to increase access to diagnosis.

## 1. Introduction

Language skills have long been identified as a core deficit in Foetal Alcohol Spectrum Disorder (FASD), with prevalence estimates of language-related disorders in Foetal Alcohol Syndrome (FAS) being between 67.2 and 81.8% [1]. Despite this, there is limited literature on the best way to assess this in a clinical setting. Additionally, with FASD prevalence rates in the UK currently estimated as between 1.8 and 3.8% [2], there is increasing need to understand how to best assess various neurocognitive domains where multidisciplinary teams are not available.

Research has shown that prenatal alcohol exposure can affect a range of language and communication areas in FASD, especially expressive and receptive language [1,3,4,5], verbal fluency and learning [6,7], reading, spelling, phonological awareness [8,9], grammar [10] and social communication [11].

Since the publication of National Institute for Health and Care Excellence (NICE) quality standards [12], UK clinical processes for assessment of FASD are developing beyond specialist clinics. Therefore, it is important to identify which tools are useful in different settings. The Scottish Intercollegiate Guidelines Network (SIGN156) guidance describes the need for a multidisciplinary assessment to diagnose FASD [13] and recommends direct clinic-based assessment where possible; however, not everyone will have access to a multidisciplinary team or teams who have suitably qualified clinicians who can carry out a direct language assessment. As the UK National FASD Clinic collects non-direct informant information, as well as completing direct testing, we are able to compare the validity of the CCC and VABS to the CELF to see if they are able to provide us with comparable information.

This paper focuses on the communication domain, with the aim of identifying the ability of standardised communication measures, which were designed for different contexts and purposes, could still hold utility. The Clinical Evaluation of Language Fundamentals (CELF) [14] and the Children’s Communication Checklist (CCC) [15], alongside the communication domain of the Vineland Adaptive Behaviour Scales (VABS) [16,17] were chosen to assess the efficacy of these domains in assessing language and communication in Foetal Alcohol Spectrum Disorder (FASD). As these tools evaluate different areas of language, we hoped to evaluate which tools are most effective at assessing different parts of language and communication. Additionally, it was questioned whether non-face-to-face tools, such as the CCC and VABS, can be considered sufficient to demonstrate deficit in the communication domain of an FASD assessment, and therefore have clinical utility in resource limited settings where Speech and Language Therapists may not be immediately available.

## 2. Materials and Methods

The UK national FASD clinic specialises in diagnosing FASD and other comorbid disorders (such as Autism, ADHD, and Social Communication Disorder). All individuals in our database have provided informed consent to be included and for their anonymised data to be used in service evaluations and audits. This research is part of a service audit and has been registered with clinical governance.

The diagnostic process at the UK National FASD Clinic consists of a multidisciplinary, comprehensive neurodevelopmental assessment, conducted over 2 days. This includes evaluating the communication and language domain of the individual’s skills. Individuals assessed at the UK National FASD Clinic arise from all parts of the United Kingdom. After initial screening processes, the first day of assessment involves direct assessment of FASD related parameters as well as an individual’s cognitive, executive functioning, memory, and language/communication skills. A series of observational questionnaires are completed by families and schools/non-family members before informants return for a second day assessment to consider wider neurodevelopmental needs. A full description of the clinic process is described elsewhere [18,19].

The clinical cohort dataset is saved with permission of patients in a secure server and used for service evaluation, clinical improvement and exploration of approaches to assessment only. As a clinical database, however, collected over 15 years, clinical practices have changed, in part because of such audits. This means that some data sets will contain more variables than others. This in turn impacts on the type of evaluation and comparisons that can be made between data points.

Language and communication are assessed in multiple ways. This includes direct face-to-face testing as well as informant and questionnaire-based evaluations to triangulate conclusions. In person, the language assessment is conducted by a Speech and Language Therapist (SaLT) who administers, scores, and interprets the CELF as well as other pragmatic and auditory processing measures. The CELF is a professionally administered standardised assessment, used to identify language delay, and diagnose language disorder in school children and adolescents aged 5–21 years. It can be used in people with and without neurodevelopmental disorders. It assesses four aspects of language (morphology and syntax, phonological awareness, semantics, and pragmatics) and provides standard scores and percentiles for Core Language, Receptive Language, Expressive Language, Language Content, Language Memory, and Language Structure. Language Memory and Language Structure are age range exclusive indexes, with Language Structure being used for individuals aged 5 to 8 years and Language Memory being used for individuals aged 9 to 21 years. Due to these age restrictions, both the Language Memory and Language Structure indexes were excluded for our analyses.

Informant measures such as the CCC are also used. The CCC is completed by a parent or caregiver who knows the individual well. It is primarily used as a screening tool to identify children who are likely to have language impairment or that would benefit from further autism assessment. It is used in individuals aged 4–16 and consists of 10 scales, each of which measure a different aspect of language or communication (speech (scale A), syntax (scale B), semantics (scale C), coherence (scale D), inappropriate initiation (scale E), scripted language (scale F), use of context (scale G), nonverbal communication (scale H), social relations (scale I), and interests (scale J)). For each subscale, a raw score can be converted to an age-standardised score and the General Communication Composite Score can be obtained from summing A–H scale scores.

The clinic also uses other informant measures such as the Vineland Adaptive Behaviour Scale (VABS), which is completed by someone who knows the individual well. The VABS can be used to assess anyone aged 0–90 and is primarily used to aid in classifying those with intellectual or developmental disabilities/disorders, as well as give an estimate as to their functional ability as compared to actual intellectual level.

Unlike the CELF, which is typically interpreted by a Speech and Language Therapist (SaLT), the CCC and VABS can be interpreted by a wider range of clinicians. As a service audit, we aim to see whether each of these measures appear to assess the same, or different, parts of language.

As a clinical sample, measures have changed with time. Therefore, not all people that came to the clinic between the years 2009 and 2025 will have the same measures, as they have been updated with time and with changes to the clinical process over that time period.

Whilst around 10% of the individuals who present to clinic are aged above 16, this evaluation is restricted to an analysis of individuals aged 16 or below. The clinic does not see children until the age of 6 in order to maximise the validity of assessments conducted.

At the time of analysis, the clinic database contained information from a total of 385 individuals. From this total, 279 individuals were aged between 6 and 16 and had received a diagnosis of FASD. Of this sample, 77 individuals had a fully completed CELF, 189 had a fully completed CCC, and 274 had a fully completed VABS. A total of 62 individuals had a fully completed CELF, CCC, and VABS. The higher number of individuals with a fully completed VABS is due to the length of time the clinic has been using the tool.

Advice was sought from our Speech and Language Therapist about which domains are most similar to those assessed by the CELF. It was advised that the A (Speech), B (Syntax), C (Semantics) and D (Coherence) domains of the CCC would correlate most closely to the CELF. The CCC-2 manual (2003) states these four scales “assess aspects of language structure, vocabulary and discourse” [15].

Data was analysed using IBM SPSS 30.0. All CELF scores were within a ±1.96 skew and kurtosis range, indicating that they were normally distributed. However, some CCC scores and the VABS Communication domain fell outside of this range, indicating that they were not normally distributed. This was in part due to the different sample sizes due to clinical data points of different tools. Due to this, percentiles were used for the CCC analyses instead of scaled scores.

Spearman’s rank correlation coefficients were used to determine relationships between the CELF, and 5 selected areas of the CCC; as well as between the CELF and the Communication domain of the VABS.

## 3. Results

All study participants met criteria for a Foetal Alcohol Spectrum Disorders diagnosis with ages ranging between 6 and 16. From the overall sample, 77 participants had a fully completed CELF (mean age = 10.48, SD = 2.81; 10 with a diagnosis of FASD with sentinel facial features, 67 with a diagnosis of FASD without sentinel facial features); 189 participants had a fully completed CCC (mean age = 10.68, SD = 2.86; 35 with a diagnosis of FASD with sentinel facial features, 154 with a diagnosis of FASD without sentinel facial features); 274 participants had a fully completed VABS (mean age = 10.63, SD = 2.96; 42 with a diagnosis of FASD with sentinel facial features, 232 with a diagnosis of FASD without sentinel facial features); and 62 participants had a fully completed CELF, CCC, and VABS (mean age 10.50, SD = 2.88; 8 with a diagnosis of FASD with sentinel facial features, 54 with a diagnosis of FASD without sentinel facial features).

When looking at IQ, scores were taken from the Wechsler Intelligence Scale for Children, Fourth Edition (WISC-IV) [20], and the Wechsler Intelligence Scale for Children, Fifth Edition (WISC-V) [21]. As participants in this dataset were seen before and after the clinic switched from using the WISC-IV to the WISC-V, means from both editions are reported here. Participants with a full CELF had a mean WISC-IV full scale IQ (FSIQ) of 82.4 (*n* = 17, SD = 10.9), and a mean WISC-V FSIQ of 79.9 (*n* = 45, SD = 13.1). Participants with a full CCC had a mean WISC-IV FSIQ of 80.3 (*n* = 36, SD = 17.0), and a mean WISC-V FSIQ of 78.6 (*n* = 101, SD = 13.2). Participants with a full VABS had a mean WISC-IV FSIQ of 80.5 (*n* = 55, SD = 17.40), and a mean WISC-V FSIQ of 79.5 (*n* = 118, SD = 13.1). Participants with a full CELF, CCC, and VABS had a mean WISC-IV FSIQ of 84.2 (*n* = 13, SD = 10.4), and a mean WISC-V FSIQ of 79.0 (*n* = 35, SD = 13.6).

Mean CELF scores were calculated (see Table 1, and one-sample *t*-tests were run to determine whether the mean CELF scores in this sample were statistically different from the CELF normative mean score of 100. All CELF scores in this sample were significantly lower than the CELF normative mean score (see Table 1).

A series of paired-sample *t*-tests with Bonferroni correction (α = 0.008) were also conducted to compare the CELF scores. Receptive Language was significantly lower than Expressive Language t(76) = −3.09, *p* = 0.003; and Receptive Language was also significantly lower than Language Content t(76) = −3.25, *p* = 0.002. No other comparisons were significant.

**Figure 1:**
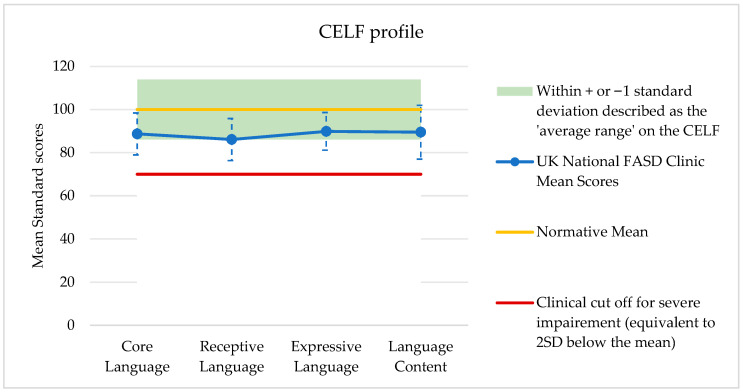
Mean Standard scores for each of the 5 CELF domains, with standard deviation ranges (*n* = 77). Normative mean is 100. Standard scores of 85 or below are identified as “mild language impairment” according to the CELF manual. A standard score of 70 or below is identified as severe impairment.

Median CELF scores were calculated and are shown in Figure 1 and Table 2. All scores fell below the normative mean but were within the Average Range. None of the Median Standard scores fell below the clinical cut-off for severe impairment, classified as 2 standard deviations below the mean; however, the interquartile ranges show the variability of scores, with the 25th percentiles of all standard scores falling in the low average range, and the 75th percentile of Language Content falling above the normative mean.

**Figure 2:**
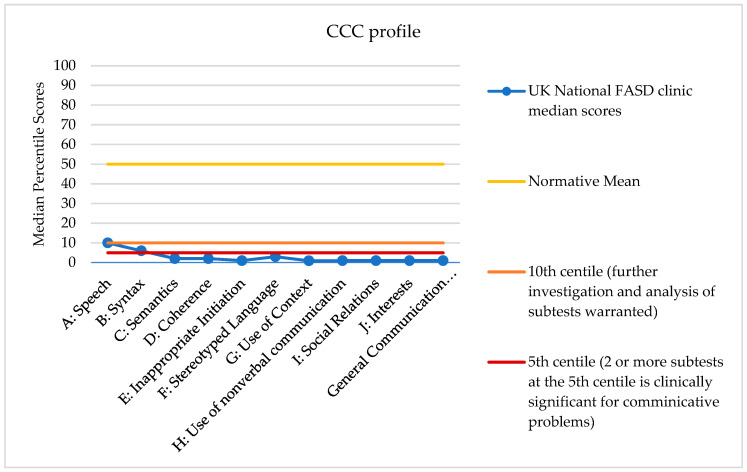
Median percentile scores for each of the CCC subscales (*n* = 189). Also shows the General Communication Composite percentile. Percentile normative mean is 50.

The median CCC percentile scores were also calculated (see Table 1 and Figure 2) and one-sample *t*-tests were run to determine whether the CCC scores in this sample were statistically different from the CCC normative mean score of 50. All CCC percentile scores in this sample were significantly lower than the CCC percentile normative mean score (see Table 1).

The median scores all fell below the normative mean of 50, and outside the average range. Every subscale fell at or below the 10th centile, with the majority also falling below the 5th centile (with the exception of A: Speech and B: Syntax). According to the CCC manual, scores falling at the 10th centile indicate that further investigation and analysis of subtest is warranted; two or more subtests at the 5th centile is clinically significant for communicative problems [15].

**Figure 3:**
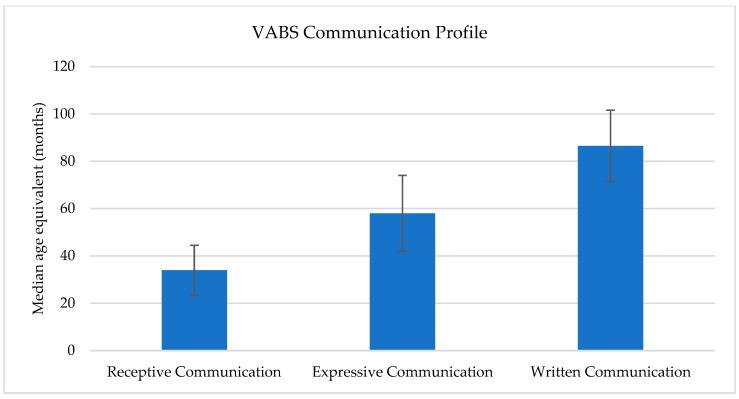
VABS communication subdomain median age equivalents (in months; *n* = 274). Median chronological age = 120.0 months (10.0 years).

Table 2 and Figure 3 show the three communication subdomains of the VABS in age equivalents (in months), in a sample with a median chronological age of 10.0 years. The median age equivalents are shown with their interquartile ranges. The means and ranges in months are shown in Table 2.

Two-tailed Spearman’s Rho Correlations between 4 CCC subscales and the GCC and all CELF domains were carried out (see Table 3). Significant positive correlations were found between the GCC of the CCC and all CELF domains; as well as between all CELF domains and the CCC Semantics and CCC Coherence domains. Significant positive correlations were also found between the CCC Syntax domain and the Core Language (*r_s_* (61) = 0.262, *p* = 0.038), Receptive Language (*r_s_* (61) = 0.249, *p* = 0.049), and Expressive Language (*r_s_* (61) = 0.317, *p* = 0.011) CELF domains. No significant correlations were found between the CCC Speech domain and any of the CELF domains, or between the CCC Syntax domain and the CELF Language Content domain (*r_s_* (61) = 0.196, *p* = 0.123).

Two-tailed Spearman’s Rho Correlations between CELF domain standard scores and VABS communication subdomain age equivalents (in months) and Communication domain standard score were also carried out (see Table 4). Significant positive correlations were found between the VABS Written Communication subdomain and all CELF domains; between the VABS Expressive Communication subdomain and CELF Core language (*r_s_* (74) = 0.240, *p* = 0.037), CELF Expressive Language (*r_s_* (74) = 0.263, *p* = 0.022), and CELF Language Content (*r_s_* (74) = 0.255, *p* = 0.026); and between the overall VABS communication domain and CELF Core Language (*r_s_* (74) = 0.264, *p* = 0.021), CELF Expressive Language (*r_s_* (74) = 0.231, *p* = 0.045), and CELF Language Content (*r_s_* (74) = 0.245, *p* = 0.03). No significant correlations were found between the VABS Receptive Communication subdomain and any of the CELF domains; between the VABS Expressive Communication subdomain and CELF Receptive Language (*r_s_* (74) = 0.156, *p* = 0.180); or between the overall VABS Communication domain and CELF Receptive Language (*r_s_* (74) = 0.217, *p* = 0.060).

## 4. Discussion

This paper identified communication profiles in a young UK FASD population, as well as finding significant relationships between language domains measured by the CELF, CCC and VABS.

Whilst the mean CELF Index scores of our sample all fell in the lower half of the average range (defined as standard scores between 86 and 114), all scores were found to be significantly lower than the normative mean of 100. Receptive Language was also found to be significantly lower than Expressive Language and Language Content in this sample, which is consistent with previous studies, which have found CELF-5 Receptive Language scores to be lower than mean CELF-5 Expressive Language scores in groups that have experienced alcohol exposure during pregnancy [22].

Whilst the mean CELF Index scores in our sample were all significantly different from the population mean, they were not below the clinical cut-off. However, the CCC median scores were routinely below the clinical cut-off (two or more subtest scores below the 5th percentile) indicating that the CCC is yielding more clinically significant communicative problems compared to the CELF.

When looking at correlations between domains being measured by the CELF and the CCC, the mix of significant correlations found suggested that these are two measures assessing similar, although not the same, aspects of language and communication. All the CELF domains were significantly correlated with the General Communication Composite (GCC) score of the CCC which suggests that overall information from parents gathered by the CCC (a standardised communication questionnaire) is correlated with how children are presenting on the CELF (a standardised clinic based direct assessment). However, there was lack of a significant correlation between the CELF domains and the CCC Speech subdomain, which indicates these measures are not necessarily fully interchangeable. There was also a high correlation between two of the structural CCC subtests (Semantic and Coherence) and all CELF domains.

There were also significant correlations found between the overall Communication domain of the VABS and the CELF Core Language, Expressive Language and Language Content domain, however the two instruments were not fully correlated at a subdomain level (e.g., the Receptive Language subdomain of the VABS was not correlated with the Receptive Index of the CELF). It is suggested that this is because the VABS is assessing functional communication whilst the CELF is assessing structural language skills, and therefore the two measures are looking at different aspects of language and its usage. The functional language deficits identified by the VABS, therefore, from a diagnostic point of view, may be better considered as evidence of impairment in the adaptive behaviour domain rather than evidence of impairment in the language domain.

There is increasing need to find validated methods to gather sufficient information in an effective manner both diagnostically and to support management for individuals with FASD. This work would suggest that the use of measures such as the CCC to supplement a diagnostic evaluation of the language domain is appropriate. It can be argued that the CCC can be usefully used by single clinician or team without access to a Speech and Language Therapist (SaLT), in order to identify language difficulties in someone with FASD. In a resource limited environment such as the NHS, or where there are difficulties in recruiting SaLTs to teams, structural CCC scores and the GCC score and can be considered as an alternative approach to measuring the language domain when is not possible for a skilled practitioner to carry out a direct language assessment.

These results also raise a question about whether the direct assessments such as the CELF are the best tool to recommend for language assessments in FASD. The CELF results did not show the same level of language deficits that are shown by the VABS and CCC. This is not surprising as the CCC and VABS are looking at the individual’s ability to communicate in day-to-day life, whereas the CELF is looking at optimal language ability in a formal setting. Whilst direct assessments are often seen as the gold standard, these results suggest that use of the direct assessment alone may miss wider functional language difficulties. In addition, the time limited nature of most clinical practice means direct assessments such as the CELF cannot always be conducted, and indirect assessments such as the CCC and VABS may be more manageable.

Further research is needed in other samples to identify if the CCC is sensitive enough to measure impairment in the language domain, and whether its routine clinical use as part of FASD assessments can help avoid potential negative clinical outcomes that could occur if language difficulties are not identified and supported. Further research into the assessment of wider language and communication difficulties, such as auditory processing difficulties, in those with FASD is also key, to best identify and support this area of difficulty that has previously been identified as a core deficit [1].

There were limitations identified in the approach to this work. Whilst this paper looked at the Composite and Major subdomains of the CELF, our database does not include the subtest scores, limiting our abilities to look at subtleties the CELF may present. Access to subtest scores such as word classes and word definitions would allow for more intricate analysis of the correlations between the CELF, CCC and VABS, and may allow for more exploration around the nature of the relationships.

It is also important to recognise that measures such as the CCC are normed from typical populations, whilst these analyses focused on a clinical sample. This has meant our data was heavily skewed. The interpretation of this should be confirmed in FASD research cohorts to replicate findings.

A clinical limitation to some of the findings presenting an immediate but unavoidable bias is that the SaLT assessment is the last session of a long clinic day. Therefore, the individual may be tired and not fully complete the CELF, even with the structural support offered to keep engagement high. However, findings are not interpreted if the SaLT does not consider them a valid representation of optimal ability. Clinical decisions are made about the suitability of tests on a case-by-case basis, and which will be most beneficial with the individual on the day. The CCC was introduced more recently and is sometimes not fully completed or sent back by the informant to seek clarifications.

Additionally, whilst it is clear from the current CCC and previous studies [22] that social communication difficulties including semantic and pragmatic deficits are seen in people with FASD, the CELF scaled scores that are most related to semantic deficits were not separately analysed and the pragmatic component of the CCC (E–J) were not analysed in this study.

Finally, due to the changing assessment measures used throughout the clinic’s history, we only had a small sample with fully completed CELFs, CCCs and VABSs (*n* = 62). Within this sample, only eight met criteria for a ‘FASD with Sentinel Facial Features’ diagnosis, meaning it was not considered statistically valid to compare results between those with FASD with Sentinel Facial Features and those with FASD without Sentinel Facial Features.

As mentioned above, the lack of access to subtest scores of the CELF means further analysis could not be carried out between CELF subtest scores and CCC and VABS domains. More intricate analysis between the specifics of language skills seen in these CELF subtest scores and communication profiles explored by other measures may help us understand the relationships found in this paper in more detail.

A continuation analysis between the CELF subdomains and the remaining six subscales of the CCC, which relate to pragmatics and social communication, may also reveal more detail around the correlations of profiles produced by these two measures. This paper has shown that the CCC, CELF and VABS cannot be used as interchangeable measures of communication and language in FASD, and therefore, to allow for better diagnostic access to those with whom a full Speech and Language Therapy assessment is not viable, a new assessment tool normed to a FASD population would need to be developed, ideally with a particular emphasis on the subdomains related to semantic and pragmatic deficits.

## 5. Conclusions

Overall, this paper has provided more detail into language and communication profiles in FASD, as well as how the measures used to create these profiles compare with each other. With the knowledge that language skills are a core deficit in FASD, specialist scales normed to this population need to be developed, and in areas where access to a multidisciplinary team with a SaLT to carry out a direct assessment is not viable, other tools may be key to identifying the communication support needs of those being assessed for FASD. Better tools and assessment batteries are needed to assess language in FASD and other neurodevelopmental disorders more widely, and whilst this paper has shown the current tools being used are able to reveal language deficits when used together, simpler and more cost-effective measures are needed to improve access to diagnoses.

## Figures and Tables

**Table 1:** One-sample *t*-tests between sample CELF means and normative CELF means of 100 (*n* = 77); and between sample CCC percentile means and normative CCC percentile mean of 50 (*n* = 189).

	Mean (SD)	df	t	*p*	Cohen’s d
CELF Core Language	88.7 (14.35)	76	−6.93	<0.001	−0.79
CELF Receptive Language	86.1 (14.62)	76	−8.35	<0.001	−0.95
CELF Expressive Language	89.9 (14.56)	76	−6.06	<0.001	−0.69
CELF Language Content	89.5 (15.10)	76	−6.12	<0.001	−0.697
CCC Speech	23.1 (27.93)	188	−13.3	<0.001	−0.94
CCC Syntax	19.0 (24.93)	188	−17.1	<0.001	−1.24
CCC Semantics	9.5 (18.83)	188	−29.6	<0.001	−2.15
CCC Coherence	7.7 (14.92)	188	−38.9	<0.001	−2.83
CCC Inappropriate Initiation	6.4 (13.31)	188	−45.0	<0.001	−3.28
CCC Stereotyped Language	11.9 (20.17)	188	−26.0	<0.001	−1.89
CCC Use of Context	5.3 (14.86)	188	−41.3	<0.001	−3.00
CCC Nonverbal Communication	6.6 (12.45)	188	−47.9	<0.001	−3.49
CCC Social Relations	5.6 (12.52)	188	−48.7	<0.001	−3.54
CCC Interests	7.1 (12.52)	188	−47.2	<0.001	−3.43
CCC General Communication Composite	5.1 (11.76)	187	−52.3	<0.001	−3.82

**Table 2:** CELF: Standard Scores (*n* = 77); CCC: Scaled Scores and General Communication Composite (GCC) percentiles (*n* = 189); Vineland Adaptive Behaviour Scale: Age equivalent scores (in months) (*n* = 274).

		Minimum	Maximum	Mean (SD)	Median (IQR)
CELF:					
	Core Language	50	117	88.7 (14.35)	91.0 (19.5)
	Receptive Language	52	109	86.1 (14.62)	88.0 (19.5)
	Expressive Language	55	130	89.9 (14.56)	93.0 (17.5)
	Language Content	58	119	89.5 (15.10)	90.0 (25.0)
CCC2:					
	A: Speech	0.5	91	-	10.0 (36.0)
	B: Syntax	0.5	80	-	6.0 (26.0)
	C: Semantics	0.5	97	-	2.0 (5.0)
	D: Coherence	0.5	88	-	2.0 (5.0)
	E: Inappropriate Initiation	0.1	69	-	1.0 (4.0)
	F: Stereotyped Language	0.5	88	-	3.0 (13.1)
	G: Use of Context	0.1	97	-	0.9 (0.5)
	H: Nonverbal Communication	0.5	91	-	1.0 (4.1)
	I: Social Relations	0.5	95	-	1.0 (5.5)
	J: Interests	0.5	71	-	1.0 (4.9)
	GCC percentile	0.1	79	-	1.0 (2.5)
VABS:					
	Receptive Communication	0	201	38.5 (22.8)	34.0 (21.0)
	Expressive Communication	10	252	67.1 (39.0)	58.0 (32.0)
	Written Communication	0	264	91.8 (32.6)	86.5 (30.3)

**Table 3:** Spearman’s Rho Correlations (ρ) between participant’s CELF standard score percentiles and CCC scaled score percentiles. Only includes participants who had both full CCC and CELF, <17 years old and FASD diagnosis (*n* = 63).

	CELF Core Language	CELF Receptive Language	CELF Expressive Language	CELF Language Content
CCC Speech (A)	0.155	0.222	0.164	0.174
CCC Syntax (B)	0.262 *	0.249 *	0.317 *	0.196
CCC Semantics (C)	0.372 **	0.343 **	0.343 **	0.323 **
CCC Coherence (D)	0.357 **	0.346 **	0.336 **	0.372 **
CCC General Communication Composite	0.369 **	0.353 **	0.378 **	0.349 **

* *p* < 0.05, ** *p* < 0.01.

**Table 4:** Spearman’s Rho Correlations (ρ) between participant’s CELF standard scores and VABS communication subdomain age equivalents (in months) and Communication domain standard score. Only includes participants with a FASD diagnosis and <17 years old who had both a fully complete CELF and VABS (*n* = 76).

	CELF Core Language	CELF Receptive Language	CELF Expressive Language	CELF Language Content
VABS Receptive Communication	0.142	0.090	0.147	0.204
VABS Expressive Communication	0.240 *	0.156	0.263 *	0.255 *
VABS Written Communication	0.281 *	0.255 *	0.265 *	0.358 **
VABS Communication	0.264 *	0.217	0.231 *	0.345 *

* *p* < 0.05, ** *p* < 0.01.

## Data Availability

Clinical data is held by the FASD clinic in keeping with NHS data storage rules. It is not openly available due to NHS consent to share rules, however please contact lead author for more information.
